# Feasibility of implementing public–private partnership (PPP) in the development of hospital services and optimizing resource allocation in Iran

**DOI:** 10.1186/s12962-020-00221-z

**Published:** 2020-08-03

**Authors:** Ahmad Sadeghi, Omid Barati, Peivand Bastani, Davood Daneshjafari, Masoud Etemadian

**Affiliations:** 1 Department of Public Health, Esfarayen Faculty of Medical Sciences, Esfarayen, Iran; 2 Health Human Resources Research Center, School of Management and Medical Informatics, Shiraz University of Medical Sciences, Shiraz, Iran; 3 Department of Economics, Allameh Tabatabai University, Tehran, Iran; 4 Department of Urology, Hasheminejad Kidney Center, Iran University of Medical Sciences, Tehran, Iran

**Keywords:** Public–private partnership, Feasibility, Hospital services

## Abstract

**Background:**

In recent decades, many countries have utilized public–private partnership (PPP) as a development initiative to reform their healthcare sectors. The present study examines the feasibility of implementing public–private partnerships for development of hospital services in Shiraz, Iran.

**Methods:**

This was a descriptive study of questionnaires carried out on one of Iran’s major southern cities (Shiraz) in 2016. Research population comprised of the hospitals affiliated to the Shiraz University of Medical Sciences (SUMS), private hospitals, charities, and healthcare investors. A total of 56 participants were chosen by convenience sampling. Data were collected using a researcher-made questionnaire. The questions` range were defined from 1 to 5. Data analysis was performed in SPSS_21_ using the Mann–Whitney test, *T* test, and Chi square test at 0.05 significance level.

**Results:**

The participants from the public sector had a significantly higher level of acquaintance with the concept of PPP and significantly more inclination to participate in such projects (P < 0.05). The mean values of the determinants of successful implementation of PPPs for hospital services were presented from the public and private participants` viewpoints as follows: public sector rated the capacity-creating (2.60 ± 0.39) and the social-cultural (2.58 ± 0.40) component as having a better condition than other determinants however, the private sector rated the financial-capital (2.64 ± 0.46) as the best. Analysis of the mean scores of determinants of implementation of PPP from the viewpoint of public and private sectors showed a significant difference in their views in terms of financial-capital and social-cultural dimensions (P < 0.05).

**Conclusions:**

According to the participants, the requirements for implementation of public–private partnerships for hospital services are not properly met. For any progress to be made in this regard, Iranian authorities and policymakers should devise a new platform for attracting private participation and improving hospitals’ readiness to engage in PPP projects.

## Background

Hospitals, as the most important health care institutions, play a critical role in the quality of healthcare services provided to the community and their true performance depends on their coordination with a wide range of political, social and cultural factors [1].

In many countries, a major portion of healthcare budget is spent on hospitals so their performance and cost-effectiveness requires constant attention [1]. One on hand, many governments struggle to fund the growing public healthcare systems, and on the other hand, public hospitals, which are reliance on public funds, are under constant pressure to cut costs despite the steady growth of their expenses [2]. In response to these challenges, many governments have tried a variety of strategies like capping the costs, increasing the funds, and encouraging the private-sector to participate in service provision and financing of healthcare [3].

Since 1990, Public–Private Partnership (PPP) model has gained growing popularity as an initiative for making better use of the capabilities of both public and private sectors, and has been successfully used in several countries to reform the healthcare sector [4]. PPP is believed to be an effective solution to the challenges of the health sector in the 21st Century [5]. In practice, this approach is an attempt to apply some of the principles of the private business, including financing balance and cost-revenue control via a coherent financial management, to address some of the major problems of the public sector [6]. PPP has been defined as a risk-sharing relationship between public and private sectors with the aim of achieving the goals of public sector [7].

Some benefits of implementation of PPP in the hospital sector include: reduced state responsibility and improved state planning ability, improved standardization, improved financing and legislation, relinquishment of state’s financing responsibility, use of competition to increase performance and efficiency, improved self-management, and decentralization of decision-making through delegation of decisions to local executives [7, 8]. Past implementations of PPP model in some countries have demonstrated positive effects on the primary care, hospital care, and integrated care, as PPP has managed to reduce the cost of service provision, improve the service quality without increasing the prices, improve the service availability and population coverage, reduce the costs of construction, operation, and renovation of public hospitals, and improve their performance indicators [9, 10].

In Iran, the public healthcare sector has access to valuable resources and facilities, which includes professional and dedicated workforce, equipment and technology, data sources, and repositories of intellectual wealth that is the result of work and experience of scholars and researchers; in contrast, the Iranian private healthcare sector enjoys a high level of motivation and excellent reputation among population, which perceive this sector as the top choice for cutting-edge healthcare services [10].

The first partnership of public and private sectors in Iran’s healthcare system occurred in 2003, when a nonprofit patient support organization called the Moheb Institute started a partnership with Hashemi Nezhad public hospital in Tehran. In that partnership, the Moheb Institute agreed to invest in the hospital in exchange for long-term lease of two of its wards. The Moheb institute invested in the leased wards by repurposing the excessive office spaces and equipping them to provide quality services at a very low stay prices (non-therapeutic prices) and with support of several insurance coverages [11]. In 2008, the second phase of this partnership began with long-term lease of the parking lot of the Hashemi Nejad Hospital and construction of the Mohab Hospital at the location. At present, the institute has plans to construct a chain of hospitals using the same partnership model [12]. The achievements of implementing PPP in Hashemi Nezhad public hospital was studied by Bastani et al. [13]. These results have shown that the hospital reach to sustainable increase in the performance indicators. So according to what was said, the main Iranian PPP model characteristics can be summarized as follows: the mutual long-term benefits for both public and private sectors, reinforcing the financial resources and compensating the lack of finance in public sector, improving the management structure and performance of the hospitals, utilization of the reputation and facilities of the public hospitals by the private sector [12, 13].

Although there have been a few examples of such use of PPP model in Iran’s healthcare sector, the scope of these efforts is negligible compared to the size of public hospital system, and there is indeed a need for further development of this model for hospital management in Iran. In view of this need, the purpose of this study was to investigate whether the PPP model or other similar models can be implemented in the public hospitals of major Iranian cities like Shiraz?

## Methods

This was a descriptive study of questionnaires carried out in 2016 in one of Iran’s major southern cities (Shiraz). Research population comprised of the hospitals affiliated to the Shiraz University of Medical Sciences (SUMC), private hospitals, charities, and healthcare investors. A total of 56 participants were chosen by convenience sampling. In this regard those population members from each of the above groups who are conveniently available to participate in study were selected. In convenience sampling no inclusion criteria identified prior to the selection of subjects. All subjects are invited to participate This method is chosen because of its incredibly prompt, uncomplicated, and economical benefits [14].

Of these 56 participants, 36 were from public sector (heads of SUMC departments, presidents and senior managers of SUMC hospitals, and some members of health care management faculty at SUMC) and 20 were selected from private sector (directors of charities, healthcare investors, presidents and board members of some private hospitals in Shiraz).

The data were collected using a researcher-made questionnaire consisting of 3 sections: the first section contained 6 questions dedicated to collection of participants’ individual and organizational information. The second section of the questionnaire contained 7 questions that evaluated the participants’ knowledge and attitude with regard to the use of PPP for hospital services. The third and most important section of the questionnaire contained 43 questions that evaluated the readiness of SUMC hospitals for PPP projects. The main dimensions of the questionnaire were designed using previous qualitative publications [10, 15].

The answers of third section were based on 5-item Likert scale, ranging from very desirable (with a score of 5) to very undesirable (with a score of 1). Accordingly, the maximum and minimum mean scores of each component were 5 and 1 respectively. The mean score of 3 was considered to signify a moderate (neutral) situation; the scores higher than 3 were interpreted as the desirability, and the scores lower than 3 were considered to imply the undesirability of the situation. To check the reliability of the instrument, the pilot questionnaire was administered to a group consisting of presidents of public and private hospitals and senior experts at SUMC. After collecting the data, Cronbach’s Alpha was computed with SPSS_21_. The Cronbach’s alpha obtained from this process was 0.83, which indicated the reliability of the used instrument. To establish the validity of the questionnaire, it was shared with a group of university professors with expertise on the subject, who were asked to give feedback regarding the questions and dimensions of the questionnaire.

In the data collection stage, questionnaires were distributed and collected both personally and through electronic correspondence. For this purpose, in a warm-up meeting held among the presidents of teaching hospitals, some of the senior experts and senior managers of SUMC and representatives of the private sector (presidents of private hospitals, directors of charities, and investors) about the prospects of public–private partnership, the research team gave a representation about PPP and tries to create the same concepts about the topic, its various models, and its status in Iran and the world, and also the objectives of the study. After the representation and the subsequent Q&A session about PPP, questionnaires were distributed among participants. For some of the presidents of SUMC hospitals, senior managers of the university, and representatives of the private sector, the questionnaires were sent and received via e-mail.

The normality of data was evaluated by Shapiro–Wilk test. The acquired data was analyzed with descriptive statistics as well as Mann–Whitney test, T-test and Chi square test. All statistical analyses were performed in SPSS_21_ with P = 0.05 considered as the level of significance.

## Results

### Individual and organizational data

Of the 56 participants, 36 (64%) were from public sector and 20 (36%) were from the private sector. The mean ages of participants from the public and private sectors were 44 ± 7 year and 58 ± 7 year, respectively. The participants from the public sector had a mean work experience of 17 ± 7 year and mean management experience of 11 ± 5 year. According to the results, 31 (86%) of the participants from the public sector had a history of partnership with the private sector in providing services of various forms, and described the result of this partnership as follows: very desirable (10%), desirable (42%), moderate (32%), undesirable (13%), very undesirable (3%). The results also showed that half of the participants from the private sector had a history and experience of investing in healthcare businesses. Half (50%) of these participants described the economic returns of the healthcare businesses as moderate, 37.5% as undesirable, and 12.5% as desirable.

## Knowledge and attitude in regard to PPP

In the second section of the questionnaire, participants were asked to describe their acquaintance with the concept of PPP in healthcare and their inclination to participate in such PPP projects. According to the results, the participants from the public sector were more familiar with this concept and also were more willing to engage in PPP projects. These results are presented in Table 1.

**Table 1:** Acquaintance of public-sector and private-sector representatives with the concept of PPP and their inclination to participate in healthcare PPP projects

	High and very high	Moderate	Low and very low
Acquaintance with the concept of PPP			
Public sector (n = 36)	26 (72.22%)	10 (27.78%)	0
Private sector (n = 20)	2 (10%)	6 (30%)	12 (60%)
Inclination to participate in PPP projects			
Public sector (n = 36)	31 (86.11%)	5 (13.89%)	0
Private sector (n = 20)	2 (10%)	7 (35%)	11 (55%)

As the above results indicate, 72.22% of the participants from the public-sector stated that they are familiar with the concept of PPP, but only 10% of the representatives of the private-sector made such statement and 60% of them confessed little familiarity with the concept. Also, 86.11% of the representatives of the public sector were highly interested in establishing partnerships with the private sector, but the representatives of the private sector were much less interested in investing in the public healthcare (10%).

Overall, the level of acquaintance with the concept of PPP and inclination to participate in such projects were significantly higher in the representatives of the public sector than those of the private sector (P < 0.05). These results are presented in Table 2.

**Table 2:** Mean scores of acquaintances with PPP and inclination to engage in healthcare PPP projects

Statistic	Public sector (n = 36)		Private sector (n = 20)		P-value
Variable	Mean	Standard deviation	Mean	Standard deviation	
Acquaintance with the concept of PPP^a^	4.00	0.75	2.25	0.85	0.001
Inclination to participate in PPP projects^a^	4.05	0.58	2.30	0.87	0.001

^a^Minimum mean score = 1, Maximum mean score = 5

The Chi square test was used to analyze the data regarding the areas of partnership that are of interest to the representatives of public and private sectors. The results obtained from this analysis are presented in Table 3.

**Table 3:** Preferred areas of public–private partnership from the viewpoint of public-sector and private-sector representatives

Statistic	Public sector (n = 36)			Private sector (n = 20)			P-value
Variable	Always	Sometimes	Never	Always	Sometimes	Never	
Project financing	20 (55%)	16 (45%)	0	5 (25%)	8 (40%)	7 (35%)	0.004
Design and construction of new hospitals	22 (61%)	14 (39%)	0	4 (20%)	7 (35%)	9 (45%)	0.000
Renovation and modernization of existing hospitals	23 (64%)	13 (36%)	0	3 (15%)	10 (50%)	7 (35%)	0.000
Launching of new facilities	20 (55%)	13 (36%)	0	0	12 (60%)	8 (40%)	0.002
Provision of non-clinical support services	29 (81%)	7 (19%)	0	5 (25%)	9 (45%)	6 (30%)	0.000
Provision of clinical support services	30 (83%)	6 (17%)	0	4 (20%)	8 (35%)	8 (40%)	0.000
Provision of specialized clinical services	5 (14%)	24 (67%)	7 (19%)	1 (5%)	11 (55%)	8 (40%)	0.000
Hospital management and administration	9 (25%)	16 (45%)	11 (30%)	2 (10%)	10 (50%)	8 (40%)	0.000

According to the above results, representatives of the public sectors were most interested in participation of the private sector in providing support services (clinical and non-clinical), renovating and modernizing existing hospitals, and launching new facilities. Meanwhile, they were least interested in participation of the private sector in hospital management and administration and provision of specialized clinical services. More specifically, 30% of these respondents stated that private sector should never partake in management and administration of public hospitals and 19% of them stated that there should never be a public–private partnership in provision of specialized clinical services. From the perspective of private sector representatives, the most interesting areas of partnership with the public sector were the provision of non-clinical support services and project financing.

## Feasibility of implementation of public–private partnerships

As Table 4 shows, there were 6 components for implementation of PPP from the perspective of public and private sector representatives. These components consist of policymaking, legal and regulatory issues, financial and capital allocation to the PPP projects, social-cultural variables, items (experience, incentive, specialty, etc.) that lead to create capabilities for PPP and procedures and processes of implementing PPP.

**Table 4:** Mean scores of determinants of implementation of PPP from the viewpoint of public-sector and private-sector representatives

	Public sector	Private sector	P-value
	M ± SD^a^	M ± SD^a^	
Policy making	2.18 ± 0.48	2.39 ± 0.77	0.102
Legal-regulatory	2.27 ± 0.53	2.37 ± 0.61	0.130
Financial-capital	2.30 ± 0.45	2.64 ± 0.46	0.016
Social-cultural	2.58 ± 0.40	1.86 ± 0.36	0.001
capacity creating	2.60 ± 0.39	2.31 ± 0.52	0.090
Procedural	2.41 ± 0.66	2.08 ± 0.49	0.081

^a^Minimum mean score = 1, Maximum mean score = 5

As the results of Table 4 show, the participants from the public sector viewed the “capacity creating” and “social-cultural” components as the factors with most satisfactory condition for public–private partnership. The participants from the private sector however named the “financial-capital” component as the factor with best condition for such partnership. Analysis of the mean scores of determinants of implementation of PPP from the viewpoint of representatives of public and private sectors showed a significant difference in their views in terms of “financial-capital” and “social-cultural” dimensions (P < 0.05).

## Discussion

According to the results, compared to the private sector, the public sector has more acquaintance with the concept of PPP and also more willingness to participate in such partnerships. This is contrary to the results of a study conducted in Sri Lanka, which showed that government officials had insufficient knowledge about PPP [16]. On the other hand, our study found the private sector to be apathetic toward participating in the public sector. There are many factors that make investment in the healthcare sector barely cost-effective and push the investors away to available alternatives. Two of these factors is the high uncertainty of return on investment and low profit in this area, which undermine the willingness of private investors to take risks. Another issue is the state imposition of unrealistically low prices for services, which poses a serious threat to the profit. Some believe that after privatization or delegation of state-run healthcare services to the private sector, government support fades away and subsequent delays in governmental reimbursement of provided services severely undermines the ability of contractors and investors to purchase equipment, laboratory materials, and medications, and even pay the personnel’s salaries. The government’s efforts to attract foreign investment to this area is also undercut by the same issue, as foreign investors expect some form of state guarantee before considering investment. Therefore, one of the major obstacles to attraction of private investment is the lack of guarantees regarding the steady support of the government [15].

The participants from public sector expressed strong tendency to establish public–private partnerships for provision of clinical and non-clinical support services, renovation and modernization of existing hospitals, and launching of new facilities. Meanwhile, the participants from the private sector expressed high interest in partnership for provision of support services and project financing.

In Australia, New Zealand, Canada, and most of the Europe, politicians are eager to employ the capabilities of private sector in provision of public services. In the UK, these partnerships are largely justified by the simple fact that the private sector is more efficient and cost-effective. In an international context, commentators have mostly encouraged the use of PPP because of their effects on accountability, service provision equality, and service availability [17]. In a study conducted in southern Iran, senior managers of public hospitals were found to be most interested in delegation of hospital food and nutrition services to the private sector and least interested in employing this sector for nursing services. According to this study, these managers also had moderate tendency to delegate laboratory and radiological services to the private sector [18]. The studies conducted by Young on outsourcing in the Australian healthcare sector [19] and by Tang Hsia et al. on Taiwanese hospitals [20] have also indicated the low inclination of managers to outsource clinical services.

In Iran, private sector participation in the form of PPP models can be realized in several areas. Given the chronic hospital bed shortage in Iran’s healthcare system and the old age and vulnerability of the existing hospital structures, the main priority of partnership with the private sector is the increase of hospital beds and renovation and reconstruction of existing hospitals. The statistics indicate that Iran’s healthcare system requires at least 100,000 additional hospital beds for it to reach a standard level. Creating such capacity is a huge endeavor that the government alone cannot afford to finance. But this objective can be achieved even in the short term with the help of nongovernmental sector, namely, the public institutions, foreign investment, and the domestic private sector. There are many examples of utilization of PPP model in different healthcare areas in different countries. For example, many countries have successfully used PPP models and private sector partnerships to increase the hospital bed capacity of their healthcare system (One example is the use of this approach to address hospital bed shortage in Brazil in 1999 [21]). In a survey conducted in Canada, 61% of participants believed that PPP is an appropriate approach for designing and constructing hospital and providing non-clinical services [3]. In a study carried out in Sri Lanka, most participants stated that PPP is suitable mechanism for providing clinical and non-clinical services. More specifically, some of the surveyed experts believed that even the management and investment aspects of healthcare services should be delegated to the private sector; but there were also others who believed that only non-clinical and support services should be included in such partnerships [14]. Other examples of the use of PPP to equip and modernize healthcare infrastructures can be found in France, Spain, Italy, and Portugal [22]. New Zealand, the UK and India have also had successful experiences in partnership with private sector in providing hospital support services such as housekeeping and cleaning, food and nutrition, pharmacy and office staffing, facilities maintenance and transportation. Therefore, the capacities of Iranian private sector too can be utilized to improve the performance of support services [23].

Regarding the determinants of feasibility of PPP for hospital services, the results of the study showed that the participants viewed none of the six determinants of PPP as being in desirable condition. In other words, the participants believed that the conditions for the use of PPP model in Iranian hospital sector are far from favorable. Although the representatives of the public sector regarded the determinants classified in social-cultural, capacity creating, and procedural categories to be in better condition, the participants from the private sector believed that the policy-making, legal-regularity, and financial-capital factors are in better shape for implementation of PPP.

In a study on the feasibility of PPP in the Philippine’s healthcare system, the most important factors in implementation of PPP projects were found to be the existence of mutual trust, legal and policy framework, institutional framework, contract management, and a monitoring and supervision system. The presence of a legal framework that could properly protects the interests of both public and private parties is a prerequisite for any PPP program. The 2011 Economic Intelligence Unit’s report on the environment for public–private partnerships in Asia–Pacific introduced four indicators for a valid legal framework: (i) consistency and quality of PPP regulations, (ii) effective PPP selection and decision-making, (iii) fairness/openness of bids and contract changes, and (iv) dispute-resolution mechanisms [24].

The most important factors of implementation of PPP in China and Indonesia have been reported to be the presence of suitable legal framework. For the UK, this factor is the existence of a strong and capable consortium and for Taiwan it is the stability of economic conditions [25]. In a study conducted by Spigars, it has been suggested that participation in PPP agreements would require specific legal and policy frameworks as well as proper legal and political capacity for launching and managing PPP projects in the government. In other words, the public sector must establish itself as a reliable partner with specific legal and regulatory boundaries [26]. Banzon has also suggested that legal framework is a necessity for protecting the interests of both public and private sectors, and that policies should serve as a clinical guide on how to cooperate and participate and ensure that there is a sufficient incentive for private investors [9].

Successful implementation of PPP projects and mutual trust between public and private partners requires a culture of honesty, transparency and open communication. To achieve this, the government must create a business environment that would signify its readiness to engage with the private sector and investors through and within the framework provided by national laws and regulations. Proper adjustment of loan rates, providing adequate capital guarantee, directing more facilities toward productive sectors, and reducing transaction and financing costs are known to be among the most effective ways of attracting private investment. For an investor to reach the point of considering investment in a project, one should be fairly confident about the profitability of the activity, preservation of business ownership, and safety of his/her capital. Also, investors tend to thoroughly examine the profit and loss prospects of all available projects, both long-term and short-term, before committing any capital and direct their resources to the activities that yield the highest returns throughout their lifetime. From this perspective, macroeconomic factors also affect the type of investment, in the sense that, when there is a positive economic outlook, which represents capital security and financial stability, investors tend to direct more capital towards long-term physical investments, but in the absence of such environment, they tend to invest in service, trading, and speculative activities. At the same time economic status of the country the same as unsustainability of the investments, varied and fluctuated rate of currency and the high risk rate of investment along with the political economic sanctions imposed on the country can all intensify the problems of attracting private partnership. The government is recommended to have a long term plan for moderating the risk of each of these conditions.

## Conclusions

According to our results, with the existing platform in place, Iranian private sector will not have much interest in investing and engaging with the public sector; therefore, Iranian authorities and policymakers should devise a new platform for attracting private participation and improving hospitals’ readiness to engage in PPP projects. In other words, there should be better preparations more drive in Iranian government and public sector for implementing this model of partnership. Development of a set of local criteria and indicators for evaluating the readiness of public-sector organizations to implement PPP projects is a necessity for gauging their strengths and weaknesses in this regard and their progress toward this objective. The next step will be to improve the performance of public organizations in this regard, as represented by the developed indicators, by revising government policies in order to form the grounds for the success of PPP programs in the hospital sector.

## Limitations

This study has some limitations because it was performed with a limited number of participants and the results were restricted to a certain geographical region. In another word, in the other countries, the perspectives of the public and private sector may be completely different. So this results may be generalized to those countries with the same setting like Iran. Moreover, the results of the studied can be improved applying a qualitative approach along with the quantitative one.

## Data Availability

Data entering sheets are available electronically and all the questionnaires are available in Health Human Resources Research Center, Shiraz, Iran.
